# Extragenic suppressor mutations that restore twitching motility to *fimL* mutants of *Pseudomonas aeruginosa* are associated with elevated intracellular cyclic AMP levels

**DOI:** 10.1002/mbo3.49

**Published:** 2012-11-28

**Authors:** Laura M Nolan, Scott A Beatson, Larry Croft, Peter M Jones, Anthony M George, John S Mattick, Lynne Turnbull, Cynthia B Whitchurch

**Affiliations:** 1 The ithree institute, University of Technology SydneySydney, New South Wales, 2007, Australia; 2 Australian Infectious Diseases Research Centre, School of Chemistry and Molecular Biosciences, University of QueenslandBrisbane, Queensland, 4072, Australia; 3 Institute for Molecular Bioscience, University of QueenslandBrisbane, Queensland, 4072, Australia; 4 School of Medical and Molecular Biosciences, University of Technology SydneySydney, New South Wales, 2007, Australia

**Keywords:** cAMP, tfp, type IV pili

## Abstract

Cyclic AMP (cAMP) is a signaling molecule that is involved in the regulation of multiple virulence systems of the opportunistic pathogen *Pseudomonas aeruginosa*. The intracellular concentration of cAMP in *P. aeruginosa* cells is tightly controlled at the levels of cAMP synthesis and degradation through regulation of the activity and/or expression of the adenylate cyclases CyaA and CyaB or the cAMP phosphodiesterase CpdA. Interestingly, mutants of *fimL*, which usually demonstrate defective twitching motility, frequently revert to a wild-type twitching-motility phenotype presumably via the acquisition of an extragenic suppressor mutation(s). In this study, we have characterized five independent *fimL* twitching-motility revertants and have determined that all have increased intracellular cAMP levels compared with the parent *fimL* mutant. Whole-genome sequencing revealed that only one of these *fimL* revertants has acquired a loss-of-function mutation in *cpdA* that accounts for the elevated levels of intracellular cAMP. As mutation of *cpdA* did not account for the restoration of twitching motility observed in the other four *fimL* revertants, these observations suggest that there is at least another, as yet unidentified, site of extragenic suppressor mutation that can cause phenotypic reversion in *fimL* mutants and modulation of intracellular cAMP levels of *P. aeruginosa*.

## Introduction

*Pseudomonas aeruginosa* is a Gram negative opportunistic pathogen, which grows in soil, marine environments, and infects many organisms including plants, nematodes, insects, and animals (Mahajan-Miklos et al. 2000; Rahme et al. 2000). In humans, *P. aeruginosa* causes serious, and often chronic, infections in those with cystic fibrosis and in immunocompromised individuals, such as those with AIDS, patients undergoing chemotherapy and those with severe burns (Lyczak et al. 2000; Tatterson et al. 2001). The success of this organism in various environments is attributed to its broad metabolic versatility (Stover et al. 2000) and its ability to produce many cell-associated and secreted virulence and survival factors (Van Delden and Iglewski 1998).

In *P. aeruginosa,* the expression of these factors is regulated by components of a large number of complex signaling pathways. One signaling molecule associated with regulation of virulence factors is 3′,5′-cyclic adenosine monophosphate (cAMP). In *P. aeruginosa*, cAMP is an allosteric regulator of the transcriptional activator Vfr (virulence factor regulator) (West et al. 1994), which has been shown to regulate, either directly or indirectly, more than 200 genes, including those associated with biogenesis and function of type IV pili (tfp), type III secretion systems (T3SS), flagella-mediated motility, and quorum sensing systems (Albus et al. 1997; Beatson et al. 2002; Dasgupta et al. 2002; Wolfgang et al. 2003).

As cAMP is involved in controlling such a wide range of important cellular functions, it is imperative that cAMP homeostasis is tightly controlled. In *P. aeruginosa,* cAMP is synthesized by two adenylate cyclases, CyaA and CyaB, with the latter contributing the majority of intracellular cAMP (icAMP) (Wolfgang et al. 2003; Fulcher et al. 2010). In *P. aeruginosa,* the 3′-5′ nucleotide phosphodiesterase CpdA serves to reduce the levels of intracellular cAMP by hydrolyzing the phosphodiester bond to release the biologically inactive 5′-AMP (Fuchs et al. 2010). Expression of *cpdA* is positively regulated by Vfr in response to elevated levels of icAMP (Fuchs et al. 2010).

The *P. aeruginosa* Chp chemosensory system and FimL have been shown to be involved in the modulation of icAMP levels (Fulcher et al. 2010; Inclan et al. 2011). The Chp chemosensory system shares many components in common with chemosensory systems that regulate flagella rotation in bacteria and in *P. aeruginosa* is thought to regulate the motors that control the extension and retraction of tfp to mediate a form of surface translocation termed twitching motility (Darzins 1993, 1994, 1995; Whitchurch et al. 2004). Control of icAMP levels by the Chp system appears to be mediated by the CheY-like components of the Chp system, PilG and PilH, which are thought to modulate icAMP levels by modulating CyaB activity (Fulcher et al. 2010).

The *P. aeruginosa* protein FimL is homologous to the N-terminal domain of ChpA and is required for normal twitching motility in *P. aeruginosa* strains PAO1 and PA103 (Whitchurch et al. 2005). Mutants of *fimL* have been recently shown to have reduced levels of icAMP compared with isogenic wild-type strains (Fulcher et al. 2010; Inclan et al. 2011). It has been suggested that regulation of icAMP levels by FimL may occur through posttranslational modulation of CyaB activity (Inclan et al. 2011).

We have previously reported that revertants of *fimL* mutants frequently arise that have regained near-wild-type twitching motility, presumably due to the acquisition of an extragenic suppressor mutation(s) (Whitchurch et al. 2005). Twitching-motility revertants were readily detectable as flares of twitching cells erupting from the edges of surface or interstitial colonies of *fimL* mutants (Whitchurch et al. 2005). One PAO1Δ*fimL* revertant was examined in more detail and was found to have significantly increased icAMP levels compared with the parent *fimL* mutant (Whitchurch et al. 2005).

In this study, we have characterized another five independently isolated *fimL* twitching-motility revertants and have determined that all had increased icAMP levels compared with the parent *fimL* mutant. Whole-genome sequencing of one of these strains revealed that it had acquired a loss-of-function mutation in *cpdA* that accounts for the elevated icAMP levels and restoration of twitching motility. Characterization of the other four *fimL* revertants indicates that there is at least one other site of suppressor mutation that can cause phenotypic reversion in *fimL* mutants and that is involved in modulation of icAMP levels of *P. aeruginosa*.

## Methods

### Bacterial strains, plasmids, and media

The strains used in this study and their relevant characteristics are shown in Table 1. *Escherichia coli* strain DH5α was used in all genetic manipulations and in the preparation of DNA sequencing templates, and *E. coli* S17-1 was used as the donor strain in bacterial conjugation for allelic exchange mutagenesis.

**Table 1:** Strains and plasmids used in this study

Strains	Relevant characteristic(s)	Source or reference
*Escherichia coli*		
DH5α	*recA endA1 gyrA96 hsd*R17 *thi-1 supE44 relA1* φ80 d*lacZ*Δ*M15*	Invitrogen
S17-1	*thi pro hsdR recA chr::RP4-2*	Simon et al. (1983)
*Pseudomonas aeruginosa*		
PAO1	Wild-type *P. aeruginosa* strain ATCC 15692	American Type Culture Collection
PAO1*pilA*	PAO1 with pilA inactivated by allelic displacement with a tellurite resistance cassette; Tel^R^	Klausen et al. (2003)
PAO1293-31E6	PAO1293 with mTn*5*-Tc insertion in *fimL;* Tc^R^	Whitchurch et al. (2005)
PAO1*fimL*::mTn*5*-Tc	PAO1 with same mTn*5*-Tc insertion in *fimL* as PAO1293-31E6; Tc^R^	This study
PAO1*fimL*_Rev1_	Independent twitching-motility revertant isolated from PAO1*fimL*:: mTn*5*-Tc; Tc^R^	This study
PAO1*fimL*_Rev2_	Independent twitching-motility revertant isolated from PAO1*fimL*:: mTn*5*-Tc; Tc^R^	This study
PAO1*fimL*_Rev3_	Independent twitching-motility revertant isolated from PAO1*fimL*:: mTn*5*-Tc; Tc^R^	This study
PAO1*fimL*_Rev4_	Independent twitching-motility revertant isolated from PAO1*fimL*:: mTn*5*-Tc; Tc^R^	This study
PAO1*fimL*_Rev5_	Independent twitching-motility revertant isolated from PAO1*fimL*:: mTn*5*-Tc; Tc^R^	This study
Plasmids		
pUCPSK	*P. aeruginosa-E. coli* shuttle vector; Ap^R^	Watson et al. (1996)
pGEM-T	*E. coli* cloning vector; Ap^R^	Promega
pOK12	*E. coli* cloning vector; Km^R^	Vieira and Messing (1991)
pRIC380	*P. aeruginosa* suicide vector; Ap^R^	Alm and Mattick (1996)
pSB62.4	Marker rescue clone of PAO1293-31E6 genomic DNA containing *fimL*::mTn*5*-Tc insertion site; Ap^R^, Tc^R^	Whitchurch et al. (2005)
pSB172.10	3.1-kb *Pst*I insert from pSB62.4 cloned into pOK12; Km^R^, Tc^R^	This study
pSB172.1	3.1-kb *Spe*I insert from pSB172.10 cloned into pRIC380; Ap^R^, Tc^R^	This study
pUCPCpdA	*cpdA* amplified from wild-type PAO1 and cloned into pUCPSK with *Plac*; Ap^R^	This study
pUCPCpdAL187R	*cpdA* amplified from PAO1*fimL*_Rev1_ and cloned into pUCPSK with *Plac*; Ap^R^	This study
pUCPCpdAR75G	*cpdA* amplified from PAO1*fimL*_Rev2_ and cloned into pUCPSK with *Plac*; Ap^R^	This study

*Pseudomonas aeruginosa* and *E. coli* were cultured on Luria–Bertani (LB) (Sambrook et al. 1989) broth solidified with agar at 1.5% or 1% (for twitching-motility stab assays) and grown overnight at 37°C. Cultures were grown in either cation-adjusted Mueller Hinton broth (CAMHB) or LB broth for *P. aeruginosa,* or LB broth for *E. coli,* and incubated overnight at 37°C, with shaking at 250 rpm. Light microscopy was performed with nutrient media (4 g/L tryptone, 2 g/L, yeast extract, 2 g/L NaCl) solidified with 8 g/L GelGro (ICN). Antibiotic concentrations used for selection of *E. coli* were 100 μg/mL ampicillin, 12.5 μg/mL tetracycline, and 50 μg/mL kanamycin, and for *P. aeruginosa* were 250 μg/mL carbenicillin and 200 μg/mL tetracycline.

### Recombinant DNA techniques

The preparation of plasmid DNA (Qiagen, Valencia, CA), restriction endonuclease digestion (New England Biolabs, Ipswich, MA), and ligation reactions (Promega, Madison, WI, and New England Biolabs) were carried out using standard protocols (Sambrook et al. 1989). The preparation of *E. coli* competent cells and transformations were performed as previously described (Sambrook et al. 1989). *Pseudomonas aeruginosa* competent cells were prepared by MgCl_2_ treatment and transformed as previously described (Mattick et al. 1987). *Pseudomonas aeruginosa* cells were prepared by sucrose treatment for electroporation and electroporated as previously described (Choi et al. 2006) with the following modifications: briefly, overnight 10 mL CAMHB cultures of *P. aeruginosa* were harvested by centrifugation at 3000*g* for 10 min at 4°C. The cell pellet was washed four times in 1 mL of ice-cold 300 mmol/L sucrose, and finally resuspended in 200 μL of ice-cold 300 mmol/L sucrose. Three hundred nanograms of plasmid DNA was added to 80–100 μL of electrocompetent cells, incubated at room temperature for 5 min, and then transferred to a 1-mm gap-width electroporation cuvette. After applying a pulse (settings: 25 μF; 200 Ω; 2.5 kV on a BTX 600 Electro Cell Manipulator; Harvard Apparatus Inc., Holliston, MA), the cells were added to 1 mL of CAMHB with 0.2% glucose and incubated at 37°C, shaking, for 1 h and then plated onto LB agar containing appropriate antibiotic selection.

Particular care was taken to ensure that *fimL* revertants were not inadvertently included in PAO1*fimL*::Tn*5*-Tc transformations. Vector controls and CpdA alleles were transformed using the same preparation of competent cells, and at least 10 independent colonies of PAO1*fimL*::Tn*5*-Tc transformants were chosen for further phenotypic characterizations including confirmation that the vector-control transformants had the expected abrogated twitching-motility phenotype.

### Phenotypic assays

Twitching motility was assayed using a modification of the subsurface stab assay described previously (Semmler et al. 1999). Briefly, the *P. aeruginosa* strain to be tested was stab inoculated through a 1% agar plate and after overnight growth at 37°C, the zone of interstitial biofilm expansion at the agar and petri dish interface was visualized by flooding the plate with a solution of 20% methanol and 10% acetic acid. The longest (a) and shortest (b) diameters of each interstitial biofilm were measured and the surface area calculated using the formula: area = abπ.

Microscopic analysis of twitching motility-mediated interstitial biofilm expansion on GelGro-solidified nutrient media was assayed using a modification of the slide assay described previously (Semmler et al. 1999). Briefly, 5 mL of molten Gelgro nutrient media was poured across four slides on a level surface and allowed to set. The slide was dried briefly and spotted with a small inoculum of the strain of interest taken from a fresh overnight plate culture. A 22 × 40-mm coverslip (0.13- to 0.16-mm thick) was carefully placed onto the solidified media and incubated at 37°C for 5 h. The GelGro slide was moved to the stage of an Olympus IX71 inverted research microscope fitted with phase contrast objectives. Images acquisition was performed using a FViewII camera (Olympus Corporation, Toyko, Japan) driven by AnalySIS Research Pro software (Soft Imaging Systems, Olympus).

Intracellular cAMP (icAMP) assays were performed as described previously (Fulcher et al. 2010). Briefly, strains were subcultured 1/100 from an overnight CAMHB culture into LB and incubated at 37°C with shaking until OD_600_ = 1.0. 1.5 mL of cells were harvested by centrifugation at 13,000*g* for 2 min at 4°C and washed twice with ice-cold 0.9 mol/L NaCl. Pellets were resuspended in 100 μL 0.1 N HCl and incubated on ice for 10 min with occasional vortexing to lyse the bacteria. Lysates were centrifuged at 13,000*g* for 5 min at 4°C to remove cellular debris, and the supernatant used to measure intracellular cAMP with an enzyme-linked immunosorbent assay (ELISA)-based assay (Cayman Chemical Company, Ann Arbor, MI) as per manufacturer's instructions.

### Construction of isogenic *fimL* mutant and isolation of revertant strains

A *fimL* allelic exchange mutant in PAO1 was constructed by using the sucrose counterselection system described previously (Schweizer 1992; Alm and Mattick 1996). A 3.1-kb *Pst*I fragment containing the *fimL*::mTn*5*-Tc allele from PAO2913-31E6 was subcloned from the marker rescue clone pSB62.4 into the vector pOK12. The resultant clone pSB172.10 was then digested with *Spe*I and the *fimL*::mTn*5*-Tc allele cloned into the suicide vector pRIC380 to produce pSB172.1. This vector carries the genes *sacBR,* which promotes sensitivity to sucrose, and *oriT,* which enables conjugal transfer. pSB172.1 was transformed into the *E. coli* donor strain S17-1 in preparation for mating with *P. aeruginosa* PAO1. Following conjugation, the transconjugants were plated onto 5% sucrose medium containing tetracycline to select for colonies in which the plasmid had excised while leaving the homologously recombined *fimL*::mTn*5*-Tc allele in the chromosome. Mutants were genotypically confirmed by Southern blot analysis.

### Sequencing and sequence analysis

PAO1*fimL*_Rev1_ genomic DNA was sequenced using an Applied Biosystems SOLiD System 2.0 sequencer at the High-throughput DNA Sequencing Unit, University of Otago, New Zealand. The 2 × 25-bp mate-pair format was utilized for sequencing with insert sizes between 1.5 and 2.5 kb. Approximately 34 million individual sequence tags were mapped, with an average depth of 131-fold coverage, to the reference genome of *P. aeruginosa* PAO1 (NC_002516.2) using the SOLiD System Analysis Pipeline Tool (corona lite). Fourteen high-quality SNPs were identified in PAO1*fimL*_Rev1_ relative to the *P. aeruginosa* PAO1 reference genome including three that were located in intergenic regions. In total, nonsynonymous substitutions were detected in seven protein-coding genes: PA0159, W35C (183697T>C); PA0400, A125R (4423850C>G); PA1029, M1V (1116213G>C); PA1459, G34A (1589438G>C); PA2400/PvdJ, P819A (2669175G>C); PA4341, E158D (4869855T>G); and PA4969/CpdA, L187R (5578940A>C).

The nucleotide sequences of the coding and upstream promoter regions of *cpdA*, *cyaA*, *cyaB*, *pilG*, *pilH*, and *vfr* were obtained by PCR amplification of the region of interest from the *P. aeruginosa* PAO1 genome and cloning of the amplicon into pGEM-T (or pUCPSK in the case of *vfr*). Automated DNA sequencing of the pGEM-T clones was performed by the Australian Genome Research Facility (University of Queensland, Brisbane, Queensland, Australia) and Macrogen Inc. (Seoul, Korea) using BigDye terminator chemistries. *cpdA* was amplified using the forward primer 5′-CATCGGGAACGGGCTAATG-3′ and the reverse primer 5′-GTAGACCCGCACTTCCAGCC-3′; *cyaA* was amplified using the forward primer 5′-CTGAGCGGACGGAAAGTC-3′, and the reverse primer 5′-CAGCGAGCAGGGTAATAC-3′; *cyaB* was amplified using the forward primer 5′-GCATCGGTCTCTTCTTGTTC-3′ and the reverse primer 5′-GTTTCGGCGGAGGAGTTC-3′; *pilG* and *pilH* were amplified using the forward primer 5′-TCCGGGCATTATGGATAGAG-3′ and the reverse primer 5′-AACCGCAGAGGTCCATGAT-3′; *vfr* was amplified using the forward primer 5′-GCCGGTACCCTTGACCACGAAGTGC-3′ (*Kpn*I site underlined) and the reverse primer 5′-CCTAAGCTTGTTCTTCCAGGAGCGTGG-3′ (*Hind*III site underlined). Nucleotide and predicted protein sequences were analyzed using MacVector (Oxford Molecular Group, Oxford, UK).

### Protein homology modeling

Primary amino acid alignments were carried out using ClustalW software (Thompson et al. 1994). A homology model of wild-type CpdA from *P. aeruginosa* was generated using the comparative alignment of Rv0805 from *Mycobacterium tuberculosis* and CpdA and the high-resolution (1.8 Å) structure of Rv0805 (PDB: 3IB8) from *M. tuberculosis* as the template to build the CpdA three-dimensional structure with Modeller (Sali and Blundell 1993; available at: http://www.salilab.org/modeller/).

## Results

### *fimL* revertants have increased intracellular cAMP levels

In order to further investigate the mechanism(s) of phenotypic reversion of *fimL* mutants, we isolated five independent twitching-motility revertants of the PAO1 *fimL* mutant PAO1*fimL*::mTn*5*-Tc. These *fimL* revertant strains are herein designated PAO1*fimL*_Rev1_, PAO1*fimL*_Rev2_, PAO1*fimL*_Rev3_, PAO1*fimL*_Rev4_, and PAO1*fimL*_Rev5_.

**Figure 1:**
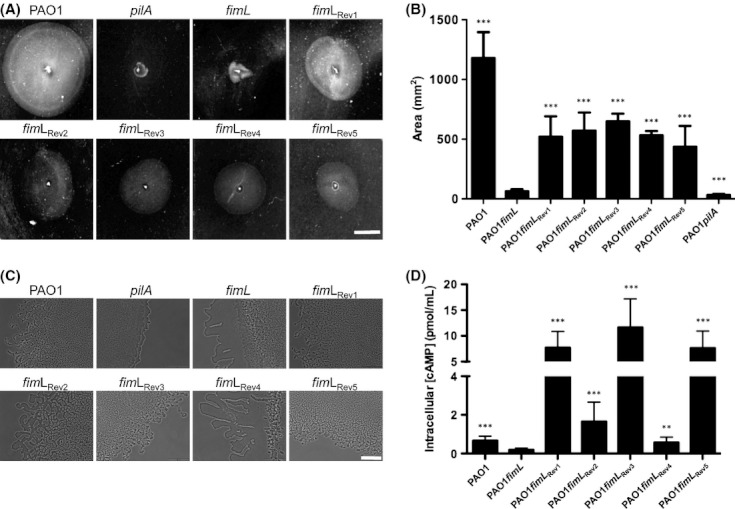
Phenotypic reversion of *fimL* mutants. (A and B) Subsurface twitching motility-mediated interstitial biofilm expansion at agar/plastic interface after 24-h incubation at 37°C. (A) Scanned images of interstitial biofilms of PAO1, PAO1*pilA*, PAO1*fimL*::mTn*5*-Tc, PAO1*fimL*_Rev1_, PAO1*fimL*_Rev2_, PAO1*fimL*_Rev3_, PAO1*fimL*_Rev4_, and PAO1*fimL*_Rev5_. Bar represents 1 cm. (B) Areas of interstitial biofilms were measured and presented as the mean ± SD from three independent experiments performed in triplicate (***P < 0.0001, Mann–Whitney *U* test compared with PAO1*fimL*::mTn*5*-Tc). (C) Light microscopy images of typical biofilm expansion at the interstitial surface between glass coverslip and Gelgro-solidified nutrient media by PAO1, PAO1*pilA*, PAO1*fimL*::mTn*5*-Tc, PAO1*fimL*_Rev1_, PAO1*fimL*_Rev2_, PAO1*fimL*_Rev3_, PAO1*fimL*_Rev4_, and PAO1*fimL*_Rev5_. Strains were incubated at 37°C for 5 h before imaging. Bar represents 100 μm. (D) Intracellular cAMP concentrations of PAO1, PAO1*fimL*::mTn*5*-Tc, PAO1*fimL*_Rev1_, PAO1*fimL*_Rev2_, PAO1*fimL*_Rev3_, PAO1*fimL*_Rev4_, and PAO1*fimL*_Rev5_. Data are presented as the mean ± SD for four independent experiments performed in triplicate (***P < 0.0001; **P < 0.005 Mann–Whitney *U* test compared with PAO1*fimL*:: mTn*5*-Tc).

The twitching-motility phenotypes of the five revertants were examined via the subsurface stab assay in which wild-type twitching motility results in rapid expansion of the interstitial biofilm that forms at the agar–petri dish interface. In this assay nontwitching mutants (such as *pilA* mutants) show no zone of biofilm expansion (Fig. 1A and B). As expected, the *fimL* mutant PAO1*fimL*::mTn*5*-Tc displayed severely abrogated twitching motility producing only a small zone of interstitial biofilm expansion via this assay (Fig. 1A and B) as described previously for a *fimL* deletion mutant of PAO1 (Whitchurch et al. 2005). Each of the *fimL* revertants demonstrated significantly greater twitching motility than the parental PAO1*fimL*::mTn*5*-Tc, although none had attained complete restoration to wild-type PAO1 levels (Fig. 1A and B).

Light microscopy was used to examine the twitching motility-mediated biofilm expansion that occurs at the interstitial interface of a glass coverslip and a pad of nutrient media solidified with gellan gum. Under these conditions, wild-type *P. aeruginosa* interstitial biofilms actively expand via tfp-mediated twitching motility and produce a characteristic micromorphological pattern that is comprised of rafts of cells at the leading edge of the biofilm, behind which forms an intricate lattice-like network of cells (Fig. 1C; Semmler et al. 1999). Nontwitching mutants, such as *pilA* mutants, demonstrate no differentiation of the biofilm edge (Fig. 1C; Semmler et al. 1999) and *fimL* mutants form large rafts of cells at the leading edge, but do not produce the intricate lattice-like network (Fig. 1C; Whitchurch et al. 2005). Microscopic examination of interstitial biofilms produced by the PAO1*fimL*::mTn*5*-Tc *fimL* revertant strains revealed obvious differences in the micromorphological patterns of the interstitial biofilms. The interstitial biofilms formed by PAO1*fimL*_Rev5_ were most similar to those formed by wild-type PAO1, except that the leading-edge rafts were routinely smaller than wild-type (Fig. 1C). The micromorphological patterns of the interstitial biofilms formed by PAO1*fimL*_Rev2_ routinely displayed wide leading-edge rafts and thicker trails immediately behind the rafts (Fig. 1C). Interestingly, the interstitial biofilms of PAO1*fimL*_Rev1_ and PAO1*fimL*_Rev3_ had similar leading-edge rafts to wild-type, but lacked the ability to form the intricate lattice-like network of trails (Fig. 1C). Instead, the cells were loosely distributed in the region behind the leading-edge rafts (Fig. 1C). Finally, the interstitial biofilms formed by PAO1*fimL*_Rev4_ appeared very similar to the parental PAO1*fimL*::mTn*5*-Tc and were comprised of extremely large, wide rafts and the cells in the regions behind the rafts were densely packed and lacked any kind of lattice-like network arrangement (Fig. 1C).

We have previously determined that a revertant of PAO1Δ*fimL* showed increased levels of icAMP (Whitchurch et al. 2005). To determine whether elevated icAMP is a phenotype common to all *fimL* revertants, we assayed icAMP levels for each of the five *fimL* revertants obtained in this study as well as the parent PAO1*fimL*::mTn*5*-Tc and wild-type PAO1 strains (Fig. 1D). We found that under the conditions of our assay, PAO1*fimL*::mTn*5*-Tc has reduced icAMP levels relative to wild-type PAO1 (Fig. 1D), as has been described previously (Inclan et al. 2011). Interestingly, each of the five *fimL* revertants showed significantly elevated levels of icAMP relative to their parent PAO1*fimL*::mTn*5*-Tc strain, although the levels of intracellular cAMP varied widely (Fig. 1D). Strains PAO1*fimL*_Rev1_, PAO1*fimL*_Rev3_, and PAO1*fimL*_Rev5_ had icAMP levels that were 10- to 20-fold higher than wild-type PAO1, whereas PAO1*fimL*_Rev2_ had icAMP levels that were only twofold higher than PAO1, and PAO1*fimL*_Rev4_ had wild-type icAMP levels (Fig. 1D). These observations suggest that extragenic suppressor mutations that lead to elevated icAMP levels are able to restore, at least in part, twitching motility to the PAO1*fimL*::mTn*5*-Tc mutant.

### *cpdA* is one site of extragenic suppressor mutation of *fimL*

To identify the site of the extragenic suppressor mutation in *fimL* revertants, we first sequenced the genome of PAO1*fimL*_Rev1_ and compared this with the available genome sequence of PAO1 (Stover et al. 2000; Winsor et al. 2011). Seven single nucleotide polymorphisms (SNPs) causing nonsynonymous amino acid substitutions were identified in the genome sequence of PAO1*fimL*_Rev1_. One of these SNPs was located in *cpdA*, which encodes a cAMP phosphodiesterase responsible for cAMP degradation in *P. aeruginosa* (Fuchs et al. 2010). Given the observed elevated icAMP levels in all of our *fimL* revertants, we considered the possibility that *cpdA* may be the site of extragenic suppressor mutation in PAO1*fimL*_Rev1_ that is responsible for the observed phenotypic reversion of twitching motility and icAMP levels. This is also consistent with the observations of Inclan et al. (2011), who found that deletion of *cpdA* restores twitching motility and T3SS function to a PAO1 *fimL* mutant (Inclan et al. 2011).

**Figure 2:**
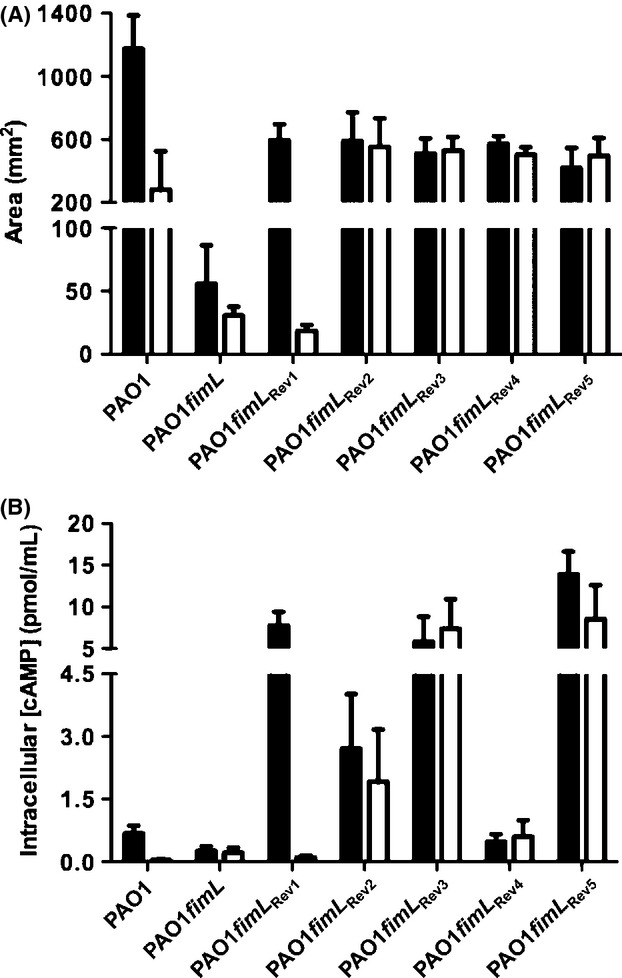
CpdA restores *fimL* phenotypes to PAO1*fimL*_Rev1_. (A) Subsurface twitching motility-mediated interstitial biofilm expansion at the agar/plastic interface. Areas of interstitial biofilms of PAO1, PAO1*fimL*::mTn*5*-Tc, PAO1*fimL*_Rev1_, PAO1*fimL*_Rev2_, PAO1*fimL*_Rev3_, PAO1*fimL*_Rev4_, and PAO1*fimL*_Rev5_ transformed with pUCPSK (solid bars) and pUCPCpdA (open bars) were measured after 24-h incubation at 37°C. Data are presented as the mean ± SD for three independent experiments performed in triplicate. (B) Intracellular cAMP concentrations of PAO1, PAO1*fimL*::mTn*5*-Tc, PAO1*fimL*_Rev1_, PAO1*fimL*_Rev2_, PAO1*fimL*_Rev3_, PAO1*fimL*_Rev4_, and PAO1*fimL*_Rev5_ transformed with pUCPSK (solid bars) and pUCPCpdA (open bars). Data are presented as the mean ± SD for four independent experiments performed in triplicate.

The SNP in *cpdA* of PAO1*fimL*_Rev1_ was confirmed by PCR amplification of *cpdA* from PAO1*fimL*_Rev1_ and sequence analysis of the cloned amplicon. The SNP in *cpdA* results in a leucine-to-arginine amino acid substitution at position 187 (L187R). To determine whether this SNP in *cpdA* is responsible for the observed increased icAMP levels and restoration of twitching motility in PAO1*fimL*_Rev1_, the plasmid pUCPCpdA (containing wild-type *cpdA*) was transformed into PAO1, PAO1*fimL*::mTn*5*-Tc, and PAO1*fimL*_Rev1_. Consistent with *cpdA* encoding a cAMP phosphodiesterase, introduction of pUCPCpdA significantly reduced icAMP levels in both wild-type PAO1 and PAO1*fimL*_Rev1_ (Fig. 2B). Interestingly, the presence of pUCPCpdA reduced twitching motility of PAO1*fimL*_Rev1_ to levels similar to those of the parental PAO1*fimL*::TcR, whereas the reduction in twitching motility was not as severe when pUCPCpdA was present in PAO1 (Fig. 2A). As introduction of the wild-type CpdA allele to PAO1*fimL*_Rev1_ restored the severely abrogated twitching-motility phenotype and icAMP to parental PAO1*fimL*::mTn*5*-Tc levels, this indicates that the L187R SNP in *cpdA* is likely to be the site of suppressor mutation in PAO1*fimL*_Rev1_.

After determining that the mechanism of reversion in PAO1*fimL*_Rev1_ is likely to be via acquisition of an extragenic suppressor mutation in *cpdA* that results in elevated icAMP levels and restoration of twitching motility_,_ we wanted to determine if *cpdA* is also the site of suppressor mutation in the other *fimL* revertants (PAO1*fimL*_Rev2_, PAO1*fimL*_Rev3_, PAO1*fimL*_Rev4_, and PAO1*fimL*_Rev5_). Interestingly, introduction of pUCPCpdA did not restore the abrogated twitching motility to any of these revertants, nor did it reduce the levels of icAMP to the same extent as when it was introduced into PAO1 or PAO1*fimL*_Rev1_. These observations suggest that *cpdA* may not be the site of suppressor mutation in these revertants and furthermore, suggest that the suppressor mutation in these strains is causing elevation of icAMP levels by a mechanism(s) that is less affected by the activity of exogenous CpdA expressed from pUCPCpdA.

We also sequenced the coding and upstream promoter regions of *cpdA* from these four *fimL* revertant strains. Sequence analysis revealed that three of the *fimL* revertants (PAO1*fimL*_Rev3_, PAO1*fimL*_Rev4_, and PAO1*fimL*_Rev5_) have wild-type *cpdA* sequences confirming that the site of extragenic suppressor mutation has occurred elsewhere in these strains. Interestingly, PAO1*fimL*_Rev2_ was found to have acquired a SNP in *cpdA,* which is predicted to result in an arginine-to-glycine amino acid change at position 75 (CpdAR75G). However, the observation that introduction of wild-type *cpdA* did not complement the revertant twitching motility and icAMP phenotypes back to parental *fimL* mutant levels suggests that this may not be the primary site of suppressor mutation in this strain or that the CpdAR75G allele is dominant negative over the introduced wild-type CpdA allele.

### CpdA alleles from *fimL* revertants have reduced activities in vivo

**Figure 3:**
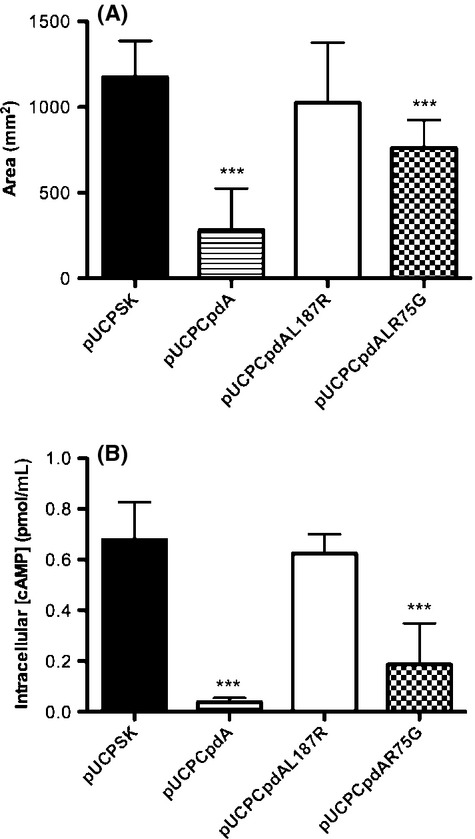
In vivo activities of CpdA alleles. (A) Subsurface twitching motility-mediated interstitial biofilm expansion at the agar/plastic interface. Areas of interstitial biofilms of PAO1 transformed with pUCPSK, pUCPCpdA, pUCPCpdAL187R, and pUCPCpdAR75G were measured after incubation at 37°C for 24 h. The data are presented as the mean ± SD from three independent experiments performed in triplicate (***P < 0.0001, Mann–Whitney *U* test compared with PAO1 transformed with pUCPSK). (B) Intracellular cAMP concentrations of PAO1 transformed with pUCPSK pUCPCpdA, pUCPCpdAL187R, and pUCPCpdAR75G. Data are presented as the mean ± SD for four independent experiments performed in triplicate (***P < 0.0001, Mann–Whitney *U* test compared with PAO1 transformed with pUCPSK).

Our results suggest that *fimL* mutants are able to restore twitching motility by acquiring extragenic suppressor mutations that lead to elevated icAMP levels, and that this increase is generated via at least two different mechanisms. In PAO1*fimL*_Rev1_ and PAO1*fimL*_Rev2_, we have identified two different SNPs in *cpdA*, which appear to affect CpdA activity, at least one of which (L187R) is likely to be responsible for phenotypic reversion in PAO1*fimL*_Rev1_. To further investigate the effect of these SNPs on CpdA activity, we cloned each of the *cpdA* genes from PAO1*fimL*_Rev1_ (pUCPCpdAL187R) and PAO1*fimL*_Rev2_ (pUCPCpdAR75G), and assayed the in vivo activity of each allele by measuring twitching motility and icAMP levels in PAO1-harboring clones expressing wild-type and mutant CpdA alleles (Fig. 3). These assays show that while pUCPCpdA significantly reduced both twitching motility and icAMP levels, pUCPCpdAL187R did not influence either twitching motility or icAMP levels suggesting that the L187R mutation leads to a loss-of-enzyme function in vivo. PAO1 containing pUPCpdAR75G demonstrated a mild reduction in both twitching motility and icAMP levels, which suggests that the R75G mutation leads to an enzyme with attenuated, but not absent, phosphodiesterase activity. Furthermore, as introduction of the CpdAR75G allele did not result in elevation of icAMP levels, these observations indicate that the CpdAR75G allele is not dominant negative over the wild-type CpdA allele present in PAO1.

**Figure 4:**
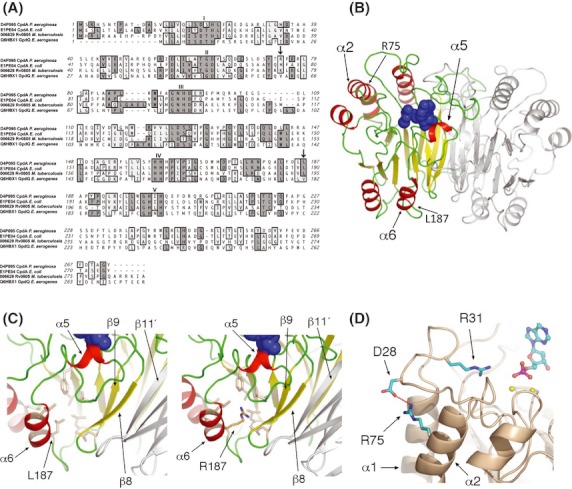
Homology modeling of CpdA alleles. (A) ClustalW alignment of *Pseudomonas aeruginosa* CpdA and homologues. ClustalW alignment of primary amino acid sequences from (top to bottom): 3′-5′-cAMP phosphodiesterase from *P. aeruginosa* (CpdA Uniprot ID: D4P095); 3′-5′-cAMP phosphodiesterase from *Escherichia coli* (CpdA Uniprot ID: E1PE04); 3′-5′-cAMP phosphodiesterase from *Mycobacterium tuberculosis* (lcc Uniprot ID: 006629); and glycerophosphodiesterase from *Enterobacter aerogenes* (phosphohydrolase Uniprot ID: Q6XBH1). The alignment was manually adjusted to fit minor mismatches. The boxed residues indicate identity (dark gray) or similarity (light gray). The five conserved, histidine-rich regions that make up the metal ion-binding sites are numbered in roman numerals. The conserved L187 and R75 are arrowed. The final 32 residues for the *M. tuberculosis* phosphodiesterase (006629) are deleted as there is not an equivalent C-terminal sequence in the other three proteins. (B) Homology model of CpdA from *P. aeruginosa* using the Rv0805 resolved structure (PDB: 3IB8) from *M. tuberculosis* as the template. The homodimer is shown with one protomer colored and the other in grayscale. Helices are in red, strands in yellow, and loops in green. 5′-cAMP was cocrystallized in the active site and is shown as space-filled blue spheres. The α-strands 4, 5, and 6 are indicated, as are the L187 and R75 residues. (C) *Pseudomonas aeruginosa* CpdA homology model showing the amino acid substitution L187R. Model depicts a close-up section of the CpdA dimer with one protomer in color and the other in grayscale. Left panel shows the conserved L187 residue and relevant secondary structural elements indicated by arrows and right panel shows the substituted R187 residue and relevant secondary structural elements indicated by arrows. (D) Model depicts a close-up section of one of the CpdA protomers. The residues D28, R31, R75, and their positively charged side chains are indicated, as are relevant secondary structural elements.

To further investigate how the L187R and R75G substitutions are likely to affect CpdA activity, we first generated a homology model of *P. aeruginosa* CpdA. Information on the structure and catalytic mechanism of *Escherichia coli* CpdA has been previously deduced by generating a homology model based on the pig purple acid phosphatase structural template (Richter 2002). We decided that a similar analysis might yield insights into the effects of the amino acid substitutions on the activity of the CpdA alleles from PAO1*fimL*_Rev1_ and PAO1*fimL*_Rev2_. The primary sequences of CpdA homologs from *E. coli*, *Enterobacter aerogenes*, and *Mycobacterium tuberculosis* were aligned with CpdA from *P. aeruginosa* using ClustalW (Thompson et al. 1994), then adjusted manually to allow for minor poor matches caused by the *E. aerogenes* GpdQ sequence (Fig. 4A). Marked on the alignment are the five highly conserved and histidine-rich metal ion-binding sites and the leucine187 or arginine75 that are substituted with arginine or glycine in PAO1*fimL*_Rev1_ and PAO1*fimL*_Rev2_, respectively. From this alignment, it is clear that leucine187 and arginine75 are highly conserved in all of these CpdA homologs, suggesting that substitutions at these positions may affect the ability of the enzyme to utilize cAMP as a substrate.

The CpdA homolog in *M. tuberculosis* is the cyclic nucleotide phosphodiesterase Rv0805, and in *E. aerogenes* is the glycerophosphodiesterase GpdQ. Crystal structures of both Rv0805 and GpdQ have been solved (Jackson et al. 2007; Shenoy et al. 2007; Podobnik et al. 2009). Although the *E. aerogenes* esterase does not hydrolyze cAMP, it does belong to the same metallo-phosphoesterase family of enzymes that hydrolyses the 3′-5′ phosphodiester bond of glycerophosphodiesters (Jackson et al. 2007). Thus, GpdQ contains the same α/β barrel metallophosphoesterase fold as Rv0805 from *M. tuberculosis*. Despite their different substrate specificities, the core and homodimer interfacial regions of GpdQ and Rv0805 are strikingly similar, with only a 2.1 Å r.m.s deviation between the dimers of the two structures (Podobnik et al. 2009).

A homology model of *P. aeruginosa* CpdA was generated using the comparative alignment of Rv0805 and CpdA (Fig. 4A). The structure of *M. tuberculosis* Rv0805 (PDB: 3IB8) was then used as the template to build the CpdA three-dimensional structure using Modeller (Sali and Blundell 1993). The rendered homology model of the complete homodimer of wild-type CpdA from *P. aeruginosa* is depicted in Figure 4B.

We then explored the predicted effects of the *cpdA* SNPs present in PAO1*fimL*_Rev1_ (L187R) and PAO1*fimL*_Rev2_ (R75G). The highly conserved L187 occurs at the C-terminus of α-helix 6, and is buried in the hydrophobic core of the protein (Fig. 4C). The L187R amino acid substitution, in placing the large and hydrophilic arginine side chain in this buried location, would likely destabilize the structural integrity of the surrounding region. This includes secondary structural elements, α5, α6, β8, and β9, which provide anchor points for loops involved in formation of the substrate-binding pocket (Fig. 4B and C). Thus, while L187 is somewhat remote from the active site, its mutation could affect function via its influence on the structure and/or dynamics of these important secondary structural elements. In addition, the hydrophobic pocket in which L187 resides also includes β-strand 9 and the loop joining it to β-strand 8, which form a significant part of the dimer interface, interacting with β-strand 11' from the opposite protomer in the homodimer (Fig. 4C). Thus, the L187R substitution could also influence enzyme function by affecting the dimer interface, the dimer being the active form of the enzyme.

R75 occurs in α-helix 2 (Fig. 4B) and may be involved in stabilizing the loop at the N-terminus of α-helix 1 through a salt bridge to D28 and a hydrogen bond to the backbone carbonyl oxygen of E27 (Fig. 4D). This loop contains R31, which is in close proximity to the phosphate group of the bound cAMP, and therefore may be involved in catalysis (Fig. 4D). The R75G substitution could therefore destabilize the geometry of the active site and effect substrate binding and/or catalysis at the active site.

### Extragenic suppressor mutations of *fimL* are not in *cyaA*, *cyaB*, *vfr*, *pilG*, or *pilH*

Our analyses have revealed that PAO1*fimL*_Rev1_ and PAO1*fimL*_Rev2_ have acquired SNPs in *cpdA* that lead to either loss of function in the CpdAL187R allele from PAO1*fimL*_Rev1_ or reduced activity in the CpdAR75G allele from PAO1*fimL*_Rev2_. The SNP present in PAO1*fimL*_Rev1_ appears to fully account for the phenotypic reversion of this strain as introduction of the wild-type CpdA allele restores the parental *fimL* mutant twitching-motility and icAMP phenotypes. Interestingly, the parental *fimL* phenotypes were not restored to any of the remaining revertants, including PAO1*fimL*_Rev2_, by introduction of pUCPCpdA, indicating that at least one other site of extragenic suppressor mutation exists in these strains. As all revertants have elevated icAMP levels, the site(s) of extragenic suppressor mutation in these strains likely occurs in a gene encoding a factor involved in controlling intracellular cAMP levels. In an attempt to identify these alternate sites of extragenic suppressor mutation, we cloned and sequenced the coding genes and upstream promoter regions of *cyaA*, *cyaB*, *vfr*, *pilG,* and *pilH* from our PAO1 strain and *fimL* revertants PAO1*fimL*_Rev2_, PAO1*fimL*_Rev3_, PAO1*fimL*_Rev4_, and PAO1*fimL*_Rev5_. No mutations were identified in any of these regions compared with the wild-type *P. aeruginosa* PAO1 reference sequence (Stover et al. 2000; Winsor et al. 2011). Therefore, the sites of extragenic suppressor mutation in these *fimL* revertants must occur within other components that are involved in modulation of icAMP levels.

## Discussion

In this study, we have characterized five independent *fimL* twitching-motility revertants and determined that each revertant has increased icAMP levels compared with the parental *fimL* mutant. Interestingly, the icAMP levels differed between the revertants, with three of the five displaying extremely high levels of icAMP. Furthermore, while macroscopic sub-surface stab assays demonstrated that each revertant had restored twitching motility-mediated interstitial biofilm expansion to almost wild-type levels, microscopic examination revealed differences in the micromorphological patterning of the expanding biofilms. Sequencing of the whole genome of PAO1*fimL*_Rev1_, identified a number of SNPs, including one in *cpdA* which encodes a cAMP phosphodiesterase. We demonstrated that this SNP (CpdAL187R) results in a loss-of-function mutation of CpdA, and is responsible for the observed increase in icAMP levels and restoration of twitching motility in PAO1*fimL*_Rev1_. We also determined that a second revertant, PAO1*fimL*_Rev2_, possesses a different SNP in *cpdA* (CpdAR75G), which likely results in reduced activity of CpdA. However, it is not clear whether this SNP accounts for the increased icAMP levels and restoration of twitching motility in PAO1*fimL*_Rev2_ as introduction of wild-type CpdA to this revertant did not restore the parental *fimL* mutant phenotypes. These observations suggested that either the SNP in *cpdA* is not the primary site of mutation responsible for the phenotypic reversion or, alternatively, that the CpdAR75G allele has a dominant negative influence over the introduced wild-type CpdA allele. However, we found that when the CpdAR75G allele was introduced into a wild-type *cpdA* background, the levels of icAMP were reduced rather than elevated. This indicates that the CpdAR75G allele possesses some cAMP phosphodiesterase activity and that this allele is not dominant negative over the wild-type allele. It is probable, therefore, that the primary site of suppressor mutation occurs elsewhere in the genome of PAO1*fimL*_Rev2_.

Interestingly, the three remaining revertants (PAO1*fimL*_Rev3_, PAO1*fimL*_Rev4_, and PAO1*fimL*_Rev5_) were found to have wild-type *cpdA* sequences indicating that the suppressor mutation has occurred elsewhere in the genomes of these strains. The observed differences in the twitching-motility and icAMP phenotypes of the five revertants are also consistent with the possibility that there are at least two mechanisms of reversion. PAO1*fimL*_Rev1_, PAO1*fimL*_Rev3_, PAO1*fimL*_Rev5_ each have very high levels of icAMP and near-wild-type twitching motility when examined microscopically, yet only PAO1*fimL*_Rev1_ has acquired a SNP in *cpdA*. Furthermore, PAO1*fimL*_Rev2_ has twofold higher icAMP levels relative to PAO1 and slightly aberrant twitching motility, whereas PAO1*fimL*_Rev4_ has wild-type icAMP levels but very aberrant twitching motility when examined microscopically.

Sequencing did not reveal any mutations in the coding and upstream promoter regions of *cyaA*, *cyaB*, *vfr*, *pilG*, and *pilH* in the *fimL* revertants, indicating that the site(s) of extragenic suppressor mutation must occur within another gene encoding a factor that is involved in control of icAMP levels in *P. aeruginosa*. The Chp chemosensory system, which has been shown to be involved in controlling icAMP levels and twitching motility (Darzins 1993, 1994, 1995; Whitchurch et al. 2004; Fulcher et al. 2010), encompasses an approximately 15 kb region. It is possible that the alternate site(s) of extragenic suppressor mutation is present within this region; however, it is also possible that there are additional components involved in controlling icAMP that are yet to be identified.

In this study, we have investigated the mechanism of twitching-motility reversion in five independent *P. aeruginosa fimL* mutants. This revealed that increasing intracellular cAMP levels is a general mechanism of twitching-motility reversion in *fimL* mutants and that this increase can occur via at least two different mechanisms. cAMP is an important second-messenger signal associated with virulence factor regulation in *P. aeruginosa*. Allosteric regulation by cAMP of the transcriptional activator Vfr (West et al. 1994) controls the expression of a large range of virulence-associated genes (Albus et al. 1997; Beatson et al. 2002; Dasgupta et al. 2002; Wolfgang et al. 2003), and it is therefore imperative that icAMP homeostasis is tightly controlled. Regulation of the adenylate cyclases, CyaA and CyaB, and the phosphodiesterase CpdA, which synthesize and breakdown cAMP, respectively, (Wolfgang et al. 2003; Fuchs et al. 2010; Fulcher et al. 2010) is mediated by a number of complex, intersecting pathways (Fulcher et al. 2010; Inclan et al. 2011), which is still not fully understood. Identification of the sites of extragenic suppressor mutation in the *fimL* mutant revertants that do not contain SNPs in *cpdA* will further increase our understanding of how icAMP levels and twitching motility are regulated to ultimately contribute to the overall virulence of *P. aeruginosa*.
